# Probabilities of evoked and spontaneous synaptic transmission at individual active zones: Lessons from *Drosophila*

**DOI:** 10.3389/fnmol.2022.1110538

**Published:** 2023-01-04

**Authors:** Maria Bykhovskaia

**Affiliations:** Neurology Department, Wayne State University, Detroit, MI, United States

**Keywords:** exocytosis, calcium, GCaMP, neuromuscular junction, Complexin, glutamate

## Abstract

Nerve terminals release neuronal transmitters at morphological specializations known as active zones (AZs). Synaptic vesicle fusion at individual AZs is probabilistic, and this property is fundamental for the neuronal information transfer. Until recently, a lack of appropriate tools limited the studies of stochastic properties of neuronal secretion at individual AZs. However, *Drosophila* transgenic lines that express postsynaptically tethered Ca^2+^ sensor GCaMP enabled the visualization of single exocytic event at individual AZs. The present mini-review discusses how this powerful approach enables the investigation of the evoked and spontaneous transmission at single AZs and promotes the understanding of the properties of both release components.

## 1. Introduction

Synaptic transmission is a probabilistic process, and stochastic properties of transmitter release are fundamental for the neuronal information transfer (Burnod and Korn, 1989; Stevens and Wang, 1994; Goda and Sudhof, 1997; Zador, 1998; Abbott and Regehr, 2004; Tarr et al., 2013). Transmitters are packaged in synaptic vesicles (SVs) and released by the fusion of SVs with the presynaptic membrane (PM). The fusion occurs at active zones (AZs), the morphological specializations seen at electron micrographs as filamentous densities surrounded the clusters of SVs (Zhai and Bellen, 2004; Van Vactor and Sigrist, 2017). SVs become attached to PM by the SNARE protein complex, and their fusion is evoked by an influx of Ca^2+^ and its binding to SV protein Synaptotagmin 1 (Syt1) (Rizo, 2022).

It was originally proposed by Bernard Katz (Katz and Miledi, 1969; Katz, 1971) that neuronal secretion can be described as a probabilistic process in which a large number of AZs enables SV fusion with low and approximately equal probabilities. Although the Katz’ model proved to be instrumental for the initial analysis of synaptic transmission (Zucker, 1973; Korn et al., 1981; Redman, 1990; Bekkers and Stevens, 1995; Quastel, 1997; Meyer et al., 2001), subsequently it became evident that several original postulates need to be revised. First, it was shown that release probabilities are highly non-uniform across AZs (Wojtowicz et al., 1994; Peled and Isacoff, 2011; Ehmann et al., 2014). Second, the analysis of release timings (Cohen et al., 1974a,b, 1981; Abenavoli et al., 2002; Leao et al., 2005) suggested that fusion events are not entirely independent. Although these spatial and temporal non-uniformities in release probabilities have been detected, it remained unclear what mechanisms control them and how do they shape neuronal networks.

Until recently, this fundamental question was confounded by the inability to accurately register fusion events at a single AZs since electrophysiology techniques typically monitor activity of an ensemble of AZs. This limitation has been overcome at the *Drosophila* larval neuromuscular junction (NMJ) employing genetic encoding of the postsynaptic fluorescent Ca^2+^ sensor GCaMP, which enabled the visualization of postsynaptic Ca^2+^ influx in response to synaptic activity (Peled and Isacoff, 2011). Subsequently, this method was modified to express membrane tethered GCaMP variants that allow robust detection of individual exocytic events (Melom et al., 2013; Peled et al., 2014; Newman et al., 2017). The present mini-review discusses how this approach enabled the investigation of the spatial and temporal heterogeneities of the release process at individual AZs.

## 2. Single SV fusion events at individual AZs can be reliably detected and resolved

**Figure 1:**
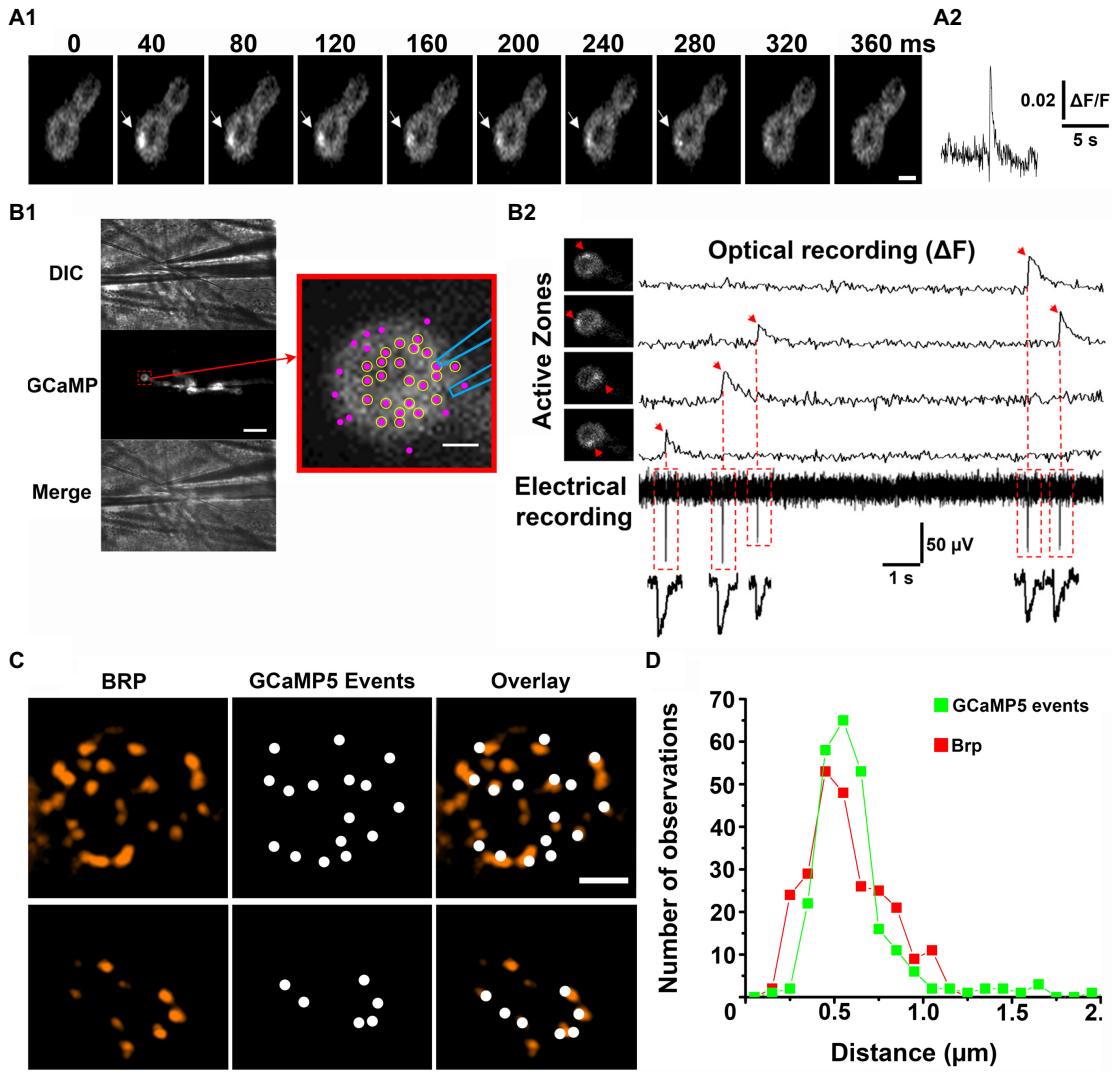
Optical detection of release events at individual AZs. Adopted from Astacio et al. (2022). **(A)** A spontaneous GCaMP5 event. Successive time frames **(A1)** and the fluorescent profile **(A2)**. **(B)** Simultaneous recordings of optical events and postsynaptic currents. **(B1)** The patch electrode of 1 μm tip diameter is positioned over the top of the bouton. Scale bar, 10 μm. The enlarged bouton shows the electrode position and the detected AZs (magenta puncta). Circles represent the AZs in which all the recorded GCaMP events had matching synaptic currents. **(B2)** Four GCaMP events produced by four different AZs (arrows) recorded simultaneously with electrical activity. The recorded synaptic currents precisely match the onsets of the recorded GCaMP events. **(C)** The detected GCaMP5 events largely co-localize with Brp puncta (two examples of synaptic boutons are shown). Scale bars: 1 μm. **(D)** The distribution of distances between the neighboring release sites defined by GCaMP events (green) versus Brp puncta (red).

It was initially shown that Ca^2+^ sensor GCaMP tethered to the inner leaflet of the postsynaptic membrane detects the postsynaptic Ca^2+^ signal produced by individual exocytic events, either evoked by an action potential or spontaneous (Melom et al., 2013). The optical events had a raising phase of tens of milliseconds and decayed within 200–300 ms (Figure 1A). The recordings of the spontaneous GCaMP signal were performed simultaneously with intracellular electrical recordings of synaptic activity, and it was shown that the fusion events recorded optically and electrically generally match (Melom et al., 2013; Newman et al., 2022). A more detailed analysis of the match between the optical and electrical events have been performed employing focal extracellular recordings (Astacio et al., 2022), and it was shown that the majority of AZs in the vicinity of the recording electrode show 100% match between the spontaneous events detected optically and electrically (Figure 1B). These findings established that the GCaMP optical signals (Figure 1A) represent single fusion events.

The next question arose of whether the spatial resolution of optical detection was sufficient to resolve fusion events at individual AZs. In other words, do optical events assigned to a single release site originate from a single morphologically defined AZ? The 3D reconstructions obtained from electron microscopy studies showed that the distances between neighboring AZs at the *Drosophila* NMJs range from 0.4 to 1.4 μm (Meinertzhagen et al., 1998). Thus, if two optical signals originate simultaneously at neighboring AZs separated by 0.4–0.5 μm they could be sometimes mistaken for a single event. However, spontaneous fusion events at individual AZs were found to be rather infrequent and typically had a frequency of less than one event per minute (Melom et al., 2013; Peled et al., 2014; Astacio et al., 2022). Therefore, it appears unlikely that two spontaneous events would originate at closely positioned AZs simultaneously. To address this question unambiguously, super-resolution microscopy was employed to investigate the co-localization of the optically detected release events with the AZ marker Bruchpilot (Brp; Kittel et al., 2006). Several studies (Akbergenova et al., 2018; Astacio et al., 2022; Newman et al., 2022) clearly demonstrated that the sites of release generally co-localized with Brp puncta (Figure 1C). To evaluate the resolution of the optical event detection, the histogram of the distances between closest neighbors for the detected GCaMP events versus Brp puncta was constructed (Astacio et al., 2022). These distributions were found to be similar (Figure 1D), suggesting that spontaneous events from individual AZs are accurately discriminated.

## 3. Spontaneous and evoked release components at individual AZs are decoupled

Spontaneous release occurs in the absence of nerve action potentials, and it was initially seen as a leak from evoked transmission (Kaeser and Regehr, 2014). However, it was shown more recently that spontaneous transmission is a distinct form of synaptic communication, which relies on a dedicated SV pool and is controlled by the molecular mechanisms that differ from those controlling evoked release (Kavalali, 2015). Are evoked and spontaneous release components segregated spatially and rely on separate pools of AZs?

It was shown that this is not the case and that numerous AZs are capable of both evoked and spontaneous fusion (Melom et al., 2013). This study demonstrated that although multiple AZs showed only one release component over the recording time, either evoked or spontaneous, approximately 40% of all the detected AZs showed both release components. A subsequent study (Peled et al., 2014) showed a strong positive correlation of the probability of evoked release with Brp fluorescence, while spontaneous events were frequently observed at AZs with low Brp levels. Finally, recent studies (Astacio et al., 2022; Newman et al., 2022) performed a systematic correlation analysis of the probabilities of evoked and spontaneous transmission at individual AZs, which were labeled by Brp and imaged with a super-resolution. These studies showed that the probabilities of the evoked and spontaneous release at individual AZs are not correlated, and that these release components are decoupled. In other words, these studies suggested that two separate mechanisms controlling spontaneous and evoked release components work independently at each AZ. Notably, a rigorous quantitative analysis of super-resolution imaging (Newman et al., 2022) revealed that these two processes are spatially segregated within an AZ, with evoked events being largely localized to the AZ center and spontaneous events predominantly occurring at AZ periphery.

Importantly, the latter study (Newman et al., 2022) also demonstrated that the decoupling between evoked and spontaneous transmission can be eliminated by the knockdown of the SNARE-associated protein Complexin (Cpx). Cpx differentially regulates the spontaneous and evoked release components, promoting evoked but inhibiting spontaneous release (Huntwork and Littleton, 2007; Jorquera et al., 2012). Notably, Cpx knockdown preparations showed a strong positive correlation between evoked and spontaneous fusion events (Newman et al., 2022). Furthermore, Cpx knockdown eliminated the spatial mismatch between the sites of evoked and spontaneous fusion within a single AZ, so that spontaneous fusion events became distributed around AZ center, similar to evoked events.

Interestingly, the evoked and spontaneous release components may be also decoupled in Syt1 deleted preparations. Indeed, the stimulation of *syt^−/−^* NMJs produced quasi-spontaneous fusion events, which were not synchronized with action potentials, but showed the distribution of activities across AZ ensemble almost identical to the distribution of the probabilities of evoked release *p_r_* (Akbergenova et al., 2018).

In summary, it was demonstrated that AZs are typically capable of both evoked and spontaneous release modes, however, these two components are controlled by distinct mechanisms and decoupled. Notably, Cpx, plays a pivotal role in decoupling the two release components. Since Cpx and Syt1 are thought to interact in the fusion process (Jorquera et al., 2012; Brunger et al., 2019), the decoupling of the two release components could be controlled by this interaction.

## 4. The probability of evoked release is largely determined by the size of the Ca^2+^ channel cluster

**Figure 2:**
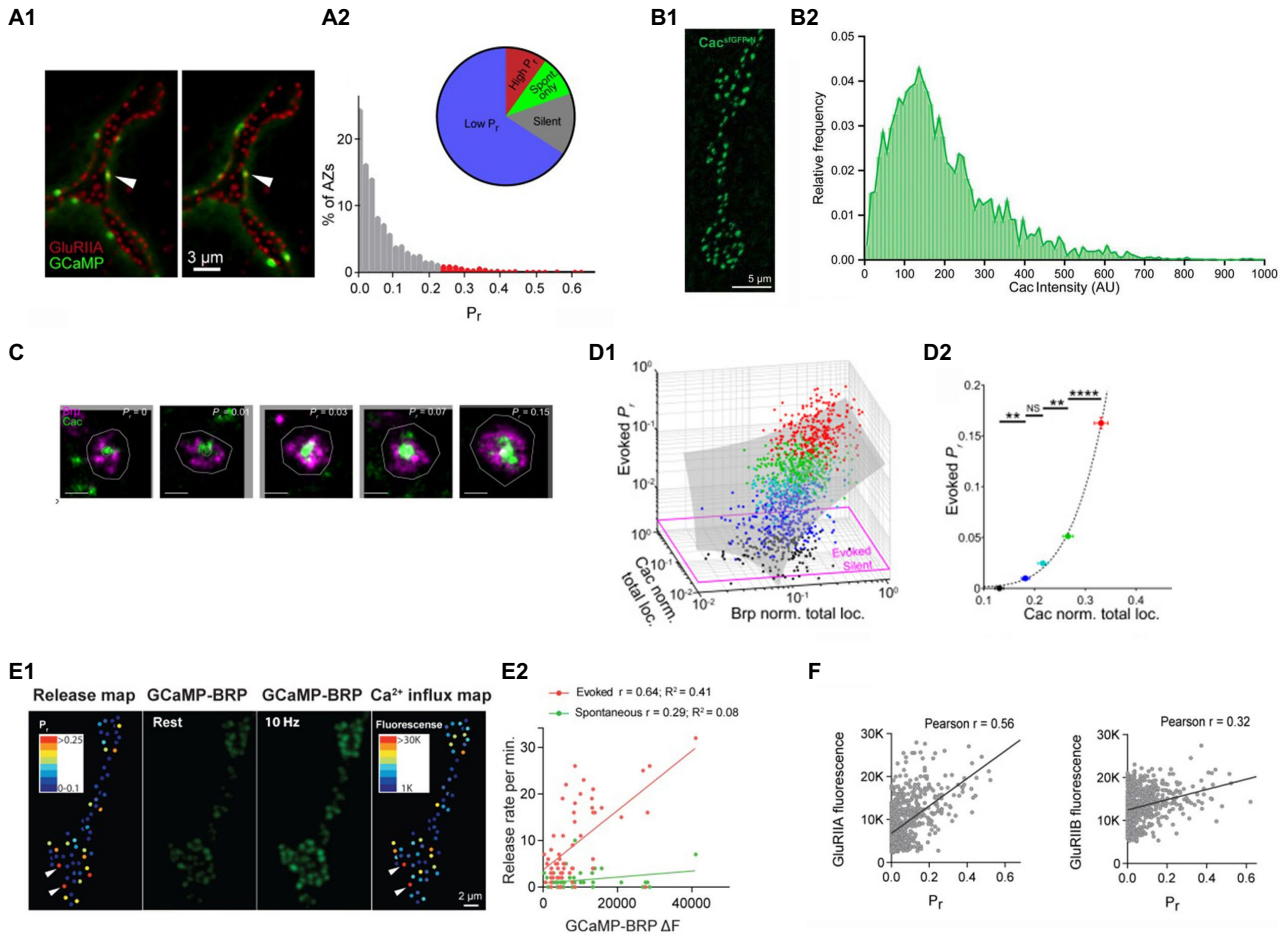
The probabilities of evoked release at individual AZs are largely determined by the clusters of Cac channels. **(A)** The distribution of the probabilities of evoked release *p_r_* is highly skewed. **(A1)** Double labeling for GCaMP events (green) and postsynaptic glutamate receptors (red). **(A2)** The distribution of *p_r_* shows a small proportion of AZs (high *p_r_*) having the release probabilities exceeding the mode of the distribution by over twenty-fold. Adopted from Akbergenova et al. (2018). **(B)** The distribution of Cac abundance across individual AZs. **(A1)** The visualization of GFP-tagged Cac clusters. **(A2)** The distribution of Cac intensity has a noticeable skew. Adopted from Gratz et al. (2019). **(C)** STORM images of Cac clusters at AZ centers (green) surrounded by Brp (red). AZs with higher *p_r_* have larger Cac clusters. Adopted from Newman et al. (2022). **(D)** The probability of evoked release *p_r_* is positively correlated with Cac abundance **(D1)** *p_r_* is positively correlated with the size of Brp and Cac clusters. **(D2)** *p_r_* dependence on Cac is super-linear (right). The data points are color-coded according to their *p_r_* values. Adopted from Newman et al. (2022). **(E)** The probability of evoked release *p_r_* is positively correlated with Ca^2+^ influx. **(E1)** The *p_r_* map and the Ca^2+^ influx map derived from the increase in GCaMP-Brp fluorescence monitored at a 10 Hz stimulation frequency. **(E2)** *p_r_*, unlike the frequency of spontaneous transmission, is positively correlated with the presynaptic Ca^2+^ signal. Adopted from Akbergenova et al. (2018). **(F)** The probability of evoked release *p_r_* shows a strong positive correlation with the abundance of GluRIIA but not GluRIIB postsynaptic glutamate receptors. Adopted from Akbergenova et al. (2018).

The distribution of the probabilities of evoked release (*p_r_*) across AZ ensemble was found to be highly skewed (Melom et al., 2013; Akbergenova et al., 2018; Gratz et al., 2019; Newman et al., 2022), with the majority of AZs showing only a single event over the observation time, while some AZs showed remarkably high activities (Figure 2A). The high *p_r_* AZs showed the release probabilities in the range of 0.2–0.7, while the mode of the distribution corresponded to 1 event over the observation time (*p_r_* < 0.01).

What makes selected AZs so efficient in generating action potential evoked release events? Since evoked fusion is triggered by Ca^2+^, it could be expected that the AZ efficacy would be affected by the magnitude of the Ca^2+^ influx and, respectively, by the abundance of the presynaptic voltage-gated Ca^2+^ channels. Notably, tagging the presynaptic voltage-gated Ca^2+^ channels Cacophony (Cac) with the fluorescent marker (Gratz et al., 2019) showed that the distribution of Cac fluorescence at individual AZs is highly skewed (Figure 2B), similar to the distribution of release probabilities. Furthermore, simultaneous imaging of Cac tagged with TdTomato (Akbergenova et al., 2018) or RFP (Gratz et al., 2019) together with the postsynaptic GCaMP signal showed a strong correlation between the release probability *p_r_* and Cac abundance.

A subsequent study (Newman et al., 2022) employed 3D-STORM super-resolution imaging to visualize AZ components and discovered that Cac clusters are located predominantly at AZ centers and surrounded by Brp (Figure 2C). This finding was in line with the discovery that the evoked events tend to originate from AZ centers, in contrast to spontaneous events. This study also showed that the probability of evoked release depends on the Ca^2+^ channel abundance in a highly non-liner manner (Figure 2D), in line with the Ca^2+^ cooperativity established for evoked release (Dodge and Rahamimoff, 1967).

The correlation between the presynaptic Ca^2+^ influx and the probability of evoked release was demonstrated using the Brp-attached presynaptic Ca^2+^ sensor GCaMP6 and the postsynaptically tethered Ca^2+^ sensor RGECO (Akbergenova et al., 2018). The presynaptic Ca^2+^ signal was reliably detected at a 10 Hz stimulation frequency, and it showed a strong correlation with the probability of evoked but not spontaneous release (Figure 2E).

Since presynaptic Cac clusters associate tightly with postsynaptic GluRIIA type receptors (Qin et al., 2005), the heterogeneity of postsynaptic glutamate receptors across AZs was also investigated (Akbergenova et al., 2018). Notably, the probability of evoked release *p_r_* showed a strong correlation with the GluRIIA receptor subtype, but not with the GluRIIB receptor subtype (Figure 2F).

Interestingly heterogeneity in *p_r_* was not reduced in Syt1 deleted NMJs, even though Syt1 deletion drastically reduced the overall probability of evoked release (Akbergenova et al., 2018). This result shows that Syt1 abundance at different SV pools and, respectively. Their Ca^2+^ sensitivity does not contribute to heterogeneity in the probabilities of evoked release, but that instead the magnitude of Ca^2+^ influx defines AZ efficacies.

Together, these studies convincingly demonstrated that the efficacies of individual AZs in generating action potential evoked release are predominantly determined by the abundance of presynaptic voltage-gated Ca^2+^ channels, which cluster tightly at AZ centers.

## 5. Spontaneous transmission is represented by a mixture of a random noise and a signaling mechanism that depends on Cpx and Ca^2+^

The distribution of frequencies of spontaneous release events at individual AZs was shown to be skewed, with the majority of AZs showing only a single event over the observation time (3–8 min), but with a small population of AZs consistently generating several events per minute (Melom et al., 2013; Peled et al., 2014). Could the high-probability spontaneous events at selected AZs result from a random variation? In the latter case, the event frequencies would obey the Poisson law, given a large number of AZs and a small release probability. Notably, it was demonstrated that only a small sub-population of AZs (10–15%) needs to be eliminated from the entire AZ ensemble in order to produce an excellent Poissonian fit for spontaneous transmission (Astacio et al., 2022). This small sub-population of AZs, however, produced a substantial proportion (approximately 40%) of all the spontaneous release events. This high activity (HA) state of an AZ was found to last typically for several minutes, and it did not correlate with AZ size. Importantly, the HA states were selectively inhibited by Cpx, and the NMJ overexpressing Cpx had the distribution of activities well fit by the Poissonian law. In contrast, the *cpx^−/−^* NMJ had the HA states selectively promoted, even though it retained the Poissonian population of events.

In addition, this study (Astacio et al., 2022) demonstrated an unexpectedly high proportion of spontaneous events that closely followed each at individual AZs. These sequences of fusion events had interevent intervals from milliseconds to hundreds of milliseconds, and they were sensitive to Ca^2+^ manipulations.

In summary, it was demonstrated that spontaneous transmission integrates a Poissonian noise with a tightly regulated signaling mechanism, which can be clamped by Cpx and enhanced by Ca^2+^ transients.

## 6. Conclusion and future directions

The optical detection of fusion events at individual AZs, which was enabled by the expression of postsynaptically tethered Ca^2+^ sensors at the *Drosophila* NMJ, greatly promoted our understanding of the probabilistic release properties. This approach unraveled the decoupling between the evoked and spontaneous release at individual AZs and enabled delineating the specific mechanisms underlying each of the two release components.

We believe this approach promises a breakthrough in understanding the release mechanisms. In particular, the role of Cpx (Newman et al., 2022) and possibly Syt1 (Akbergenova et al., 2018) in decoupling the evoked and spontaneous release components raises the question of how specifically these synaptic proteins control sorting the sites of release. Notably, tremendous progress has been achieved recently in understanding the molecular regulation of synaptic transmission (Rizo, 2022), and the major molecular components of the release machinery have been identified. The analysis of synaptic transmission at individual AZs will enable the investigation of the contribution of the synaptic machinery components to the stochastic properties of the release process, including its spatial and temporal heterogeneity and a possible interdependence of the release components and events across neighboring AZs, as well as within individual AZs.

Notably, recent studies expressed GCaMP in dendritic spines to investigate the transmission heterogeneity at individual hippocampal synapses (Metzbower et al., 2019; Durst et al., 2022). These studies demonstrated that the method application is much broader than the *Drosophila* model, and that this approach will be instrumental for understanding the function of neuronal networks in the mammalian brain.

## Author contributions

The author confirms being the sole contributor of this work and has approved it for publication.

## Conflict of interest

The author declares that the research was conducted in the absence of any commercial or financial relationships that could be construed as a potential conflict of interest.

## Publisher’s note

All claims expressed in this article are solely those of the authors and do not necessarily represent those of their affiliated organizations, or those of the publisher, the editors and the reviewers. Any product that may be evaluated in this article, or claim that may be made by its manufacturer, is not guaranteed or endorsed by the publisher.
